# A female Viking warrior confirmed by genomics

**DOI:** 10.1002/ajpa.23308

**Published:** 2017-09-08

**Authors:** Charlotte Hedenstierna‐Jonson, Anna Kjellström, Torun Zachrisson, Maja Krzewińska, Veronica Sobrado, Neil Price, Torsten Günther, Mattias Jakobsson, Anders Götherström, Jan Storå

**Affiliations:** ^1^ Archaeological Research Laboratory, Department of Archaeology and Classical Studies Stockholm University, Lilla Frescativägen 7 106 91 Stockholm Sweden; ^2^ Osteoarchaeological Research Laboratory, Department of Archaeology and Classical Studies Stockholm University, Lilla Frescativägen 7 106 91 Stockholm Sweden; ^3^ Department of Archaeology and Ancient History Uppsala University, Engelska Parken, Thunbergsvägen 3H 751 26 Uppsala Sweden; ^4^ Department Organismal Biology and Sci Life Lab Evolutionary Biology Centre, Norbyvägen 18 A 752 36 Uppsala Sweden

## Abstract

**Objectives:**

The objective of this study has been to confirm the sex and the affinity of an individual buried in a well‐furnished warrior grave (Bj 581) in the Viking Age town of Birka, Sweden. Previously, based on the material and historical records, the male sex has been associated with the gender of the warrior and such was the case with Bj 581. An earlier osteological classification of the individual as female was considered controversial in a historical and archaeological context. A genomic confirmation of the biological sex of the individual was considered necessary to solve the issue.

**Materials and methods:**

Genome‐wide sequence data was generated in order to confirm the biological sex, to support skeletal integrity, and to investigate the genetic relationship of the individual to ancient individuals as well as modern‐day groups. Additionally, a strontium isotope analysis was conducted to highlight the mobility of the individual.

**Results:**

The genomic results revealed the lack of a Y‐chromosome and thus a female biological sex, and the mtDNA analyses support a single‐individual origin of sampled elements. The genetic affinity is close to present‐day North Europeans, and within Sweden to the southern and south‐central region. Nevertheless, the Sr values are not conclusive as to whether she was of local or nonlocal origin.

**Discussion:**

The identification of a female Viking warrior provides a unique insight into the Viking society, social constructions, and exceptions to the norm in the Viking time‐period. The results call for caution against generalizations regarding social orders in past societies.

## 1 INTRODUCTION

Already in the early middle ages, there were narratives about fierce female Vikings fighting alongside men. Although, continuously reoccurring in art as well as in poetry, the women warriors have generally been dismissed as mythological phenomena (Gardeła, 2013; Jesch, 1991; Jochens, 1996).

**Figure 1:**
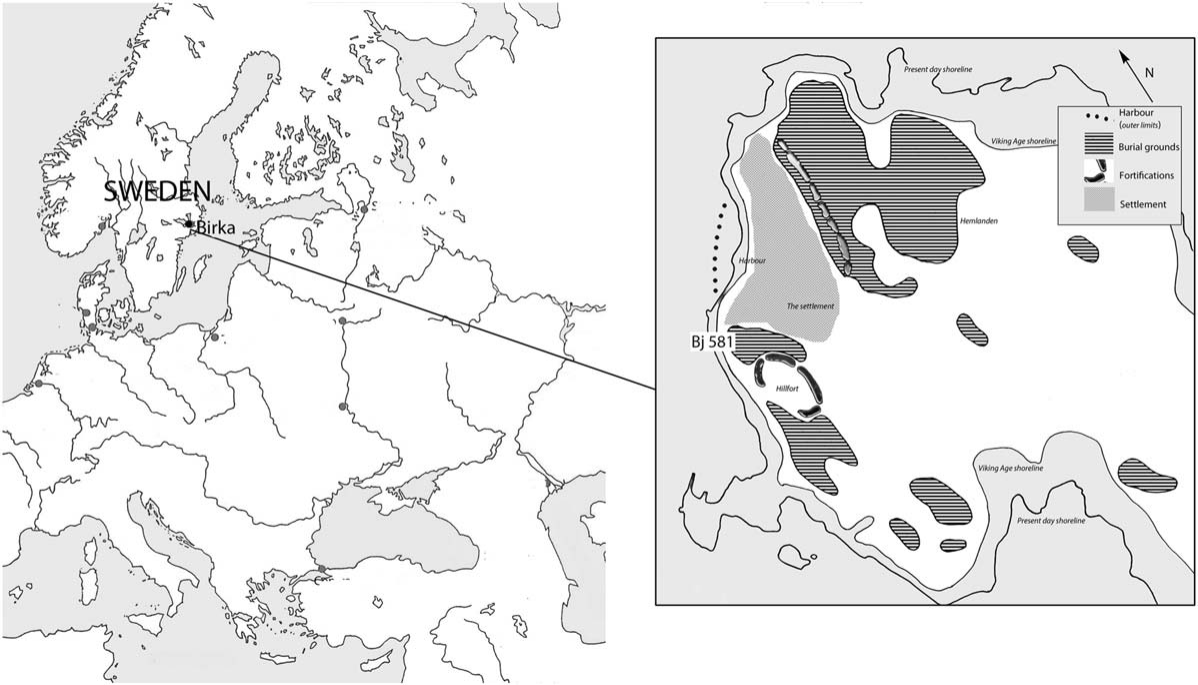
Map showing the location of Birka and grave Bj 581

Archaeological evidence of warrior graves is numerous, especially in the Viking Age of Northern Europe. Situated in Eastern Central Sweden, Birka was a key centre for trade during the 8th–late 10th century (Figure 1) (S1), linked to a social, cultural and economic network that reached beyond the Ural Mountains into the Caliphate in the east and south to the Byzantine Empire (Ambrosiani, 2012). Birka's population of approximately 700–1000 inhabitants consisted of trading families, artisans and warriors (Hedenstierna‐Jonson, 2014). The urban culture in Birka was different from the everyday life and practices of the surrounding region.

One of the strongest features reflected through the archaeological remains is the extent and diversity of contacts and cultural influences from other places (Ambrosiani, 2012; Arbman, 1941; Hedenstierna‐Jonson, 2014), which is also reflected in the diverse burial practice (Gräslund, 1980). Over 3,000 graves are known, of which approximately 1,100 have been excavated, making it one of the largest known congregations of burials in the Viking world. The graves are distributed over large burial grounds encircling the town area.

**Figure 2:**
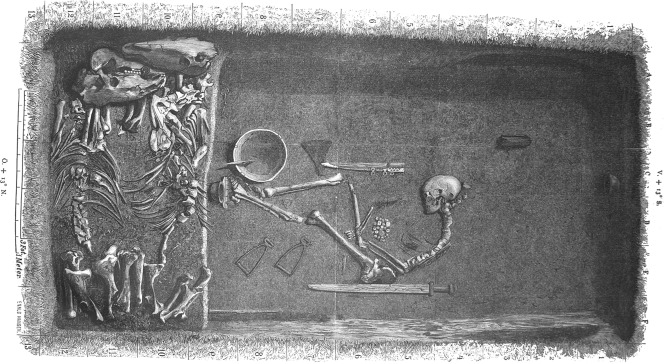
Illustration by Evald Hansen based on the original plan of grave Bj 581 by excavator Hjalmar Stolpe; published in 1889 (Stolpe, 1889)

One warrior grave, Bj 581, stands out as exceptionally well‐furnished and complete (Arbman, 1941; Thålin‐Bergman, 1986) (Figure 2 and S1). Prominently placed on an elevated terrace between the town and a hillfort, the grave was in direct contact with Birka's garrison. The grave goods include a sword, an axe, a spear, armour‐piercing arrows, a battle knife, two shields, and two horses, one mare and one stallion; thus, the complete equipment of a professional warrior. Furthermore, a full set of gaming pieces indicates knowledge of tactics and strategy (van Hamel, 1934; Whittaker, 2006), stressing the buried individual's role as a high‐ranking officer. As suggested from the material and historical records (Jesch, 1991; Jochens, 1996), the male sex has been associated with the gender of a warrior identity. Hence, the individual in Bj 581 was considered a male based on the assemblage of grave goods (Arbman, 1941; Gräslund, 1980), and the sex was only questioned after a full osteological and contextual analysis (Kjellström, 2016) that showed that the individual was a woman (S2 and S3). The existence of female warriors in Viking Age Scandinavia has been debated among scholars (Gardeła, 2013; Jesch, 1991; Jochens, 1996). Though some Viking women buried with weapons are known, a female warrior of this importance has never been determined and Viking scholars have been reluctant to acknowledge the agency of women with weapons (Hernæs, 1984; Moen, 2011) (S1). The osteological analysis triggered questions concerning sex, gender and identity among Viking warriors. This made it important to further investigate the biological sex and to do additional analyses to explore the genetic affinity of the individual buried in Bj 581. Here we present data including nuclear DNA and strontium isotopes of the individual.

## 2 MATERIALS AND METHODS

### 2.1 Osteology

The skeleton was represented by bone elements from all body regions (S2). Stored with Bj 581 was also a femur belonging to another burial which was excluded. The age and sex estimation results, presented at a conference in 2014 (Kjellström, 2016), were based on osteological standard methods for morphologic indicators (Buikstra & Ubelaker, 1994) (S2–S3). The epiphyseal union was completed on all preserved bones, and the appearance of the auricular surface of the left hip bone meets the morphologic criteria for phase 3 according to methods by Lovejoy, Meindl, Mensforth, and Pryzbeck (1985) and Meindl and Owen (1989). Furthermore, the dental wear of the lower molars was clear but moderate (stage 2–4) (Brothwell, 1981). In all, this suggests that the individual was at least above 30 years of age. The greater sciatic notch of the hip bone was broad, and a wide preauricular sulcus was present. This, together with the lack of projection of the mental eminence on the mandible, assessed the individual as female. Additionally, the long bones are thin, slender and gracile which provide further indirect support for the assessment. No pathological or traumatic injuries were observed.

### 2.2 Archaeological samples

Two samples intended for DNA analyses were removed from individual Bj 581. The samples were taken from the left canine and the left humerus; see Supporting Information Appendix, Section S3 for details.

### 2.3 DNA extraction and sequencing

All laboratory procedures were carried out at the Archaeological Research Laboratory (AFL), Stockholm University. DNA was extracted using urea‐based extraction buffer combined with silica‐based spin column purification—MinElute PCR Purification Kit (Qiagen), according to the manufactures protocols (Malmström et al., 2009; Yang, Eng, Waye, Dudar, & Saunders, 1998). The obtained DNA extracts were used for preparation of blunt‐end Illumina genomic libraries (Meyer & Kircher, 2010). The number of amplification cycles was estimated using gel electrophoresis and the libraries were amplified with AmpliTaq^®^ Gold DNA Polymerase (Applied Biosystems™) in six separate PCR reactions. The amplified products were pooled, purified with Agencourt AMPure beads (Beckman Coulter), quantified using DNA High Sensitivity Kit with Agilent 2100 Bioanalyzer Instrument (Agilent Technologies) and shotgun sequenced on Illumina HiSeq platforms at the Science for Life Laboratory Sequencing Centre in Stockholm. Raw DNA data was sorted into individual samples based on tagged sequences (de‐multiplexed), quality‐controlled and delivered to UPPNEX (UPPmax NEXt Generation sequence Cluster & Storage) (Lampa, Dahlo, Olason, Hagberg, & Spjuth, 2013).

### 2.4 Sequence analyses

**Figure 3:**
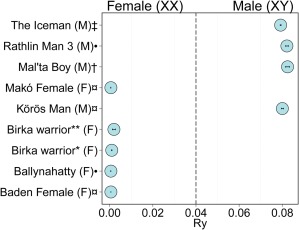
Proportion of reads aligning to chromosomes X and Y indicating biological sex in the canine (*) and humerus (**) from Birka warrior compared with sexing results from number of published ancient individuals: Cassidy et al. (2016) (•), Gamba et al. (2014) (¤), Keller et al. (2012) (‡), Raghavan et al. (2014) (†). The biological sex is given in parenthesis

The computations were performed on resources provided by SNIC through Uppsala Multidisciplinary Center for Advanced Computational Science (UPPMAX) under the following projects: b2013240, b2015307, and b2016056. Sequence data was analyzed following previously published procedures (Günther et al., 2015; Omrak et al., 2016). De‐multiplexed sequencing pair‐end reads were merged, trimmed and then mapped to the human reference genomes build 36 and 37 with BWA v. 0.7.13 (Li & Durbin, 2010). The PCR duplicates were removed with FilterUniqueSAMCons.py (Kircher, 2012). Obtained DNA fragments were then checked for presence of 3′ and 5′ degradation patterns characteristic of ancient DNA (Briggs et al., 2007, Brotherton et al., 2007, Sawyer, Krause, Guschanski, Savolainen, & Pääbo, 2012) using PMDtools (Skoglund et al., 2014). Molecular sex assignment was estimated based on the ratio of sequences aligning to the two sex chromosomes, X and Y (Skoglund, Storå, Götherström, & Jakobsson, 2013) (Figure 3, S4). Levels of contamination were estimated based on the analyses of contradictory positions in mitochondrial sequences (Green et al., 2008). The consensus mitochondrial DNA (mtDNA) sequences were called using *samtools* package (Li et al. 2009) while the initial haplogroup assignment was in HAPLOFIND tool (Vianello et al., 2013). The assignments were inspected manually by checking against PhyloTree–mtDNA tree build 17 (18 February 2016) (van Oven & Kayser, 2009). For details consult Supporting Information Appendix, Section S4 and Table S4.2.

### 2.5 Reference population panel

The genetic data from the Birka warrior was merged with three different population reference data‐sets consisting of genotype SNP data from: the Human Origins dataset (Patterson et al., 2012; Lazaridis et al., 2014), the Swedish reference (Salmela et al., 2011), and the Population Reference Sample–POPRES (Nelson et al., 2008) merged with 60 Yoruban individuals from the pilot phase of the 1000 Genomes Project (The 1000 Genome Project Consortium, 2010). The analyses were restricted to nucleotide positions with minimum mapping and base quality of 30.

### 2.6 Population comparisons

**Figure 4:**
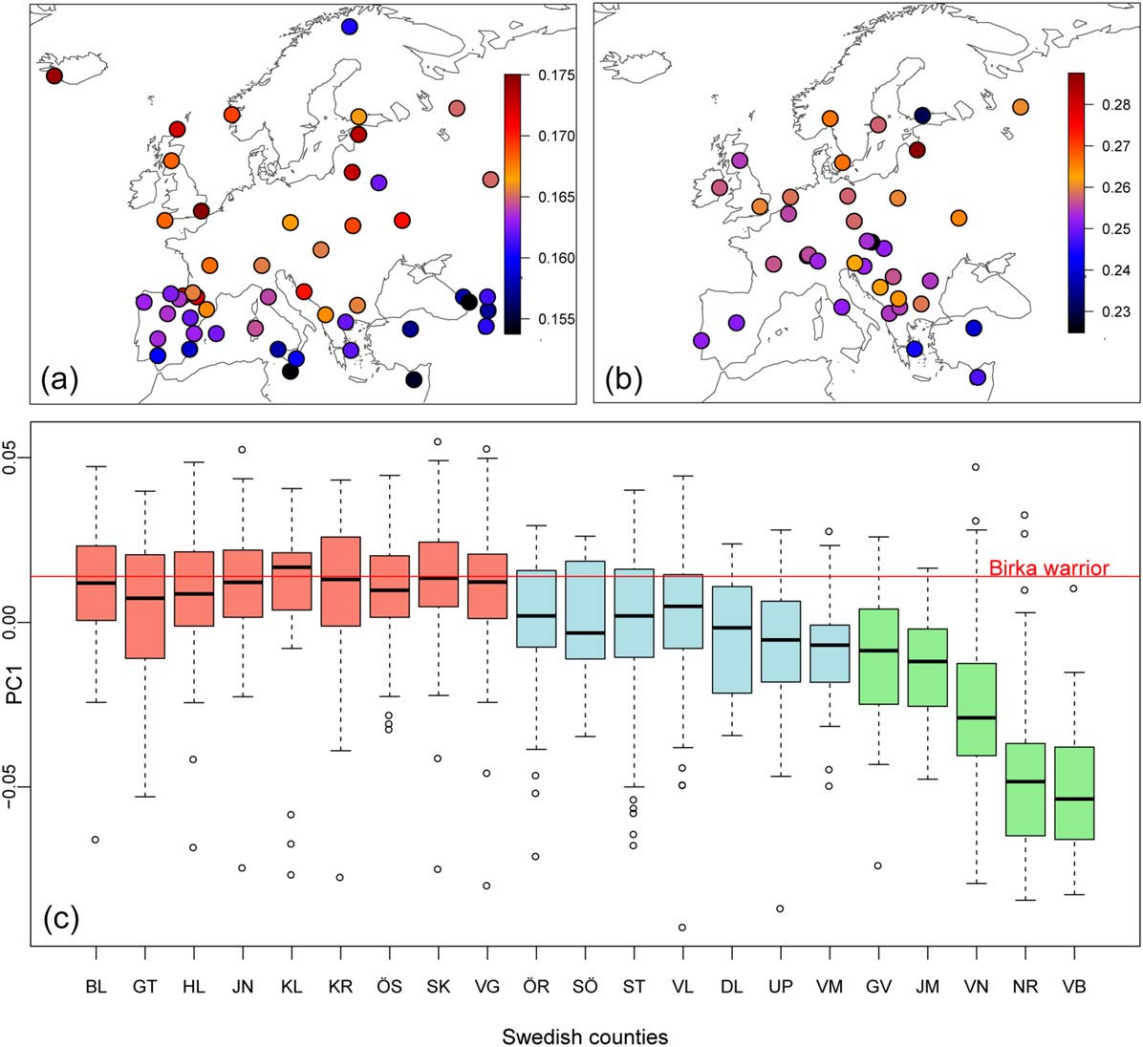
Maps visualizing the results of *f_3_*‐statistic in which the individual from grave Bj 581 was compared to (a) Human Origins population reference panel (Lazaridis et al., 2014; Patterson et al., 2012) and (b) Population Reference Sample (POPRES) (Nelson et al., 2008). (c) The Birka warrior plotted against PC1 values for 21 Swedish subpopulations representing all counties and the total of 1525 individuals (Salmela et al., 2011). The three colours represent the conventional regional division to the southern Götaland (red), central Svealand (blue), and northern Norrland (green). Abbreviations for the different counties are as follows: BL—Blekinge län, GT—Gotlands län, HL—Hallands län, JN—Jönköpings län, KL—Kalmar län, KR—Kronobergs län, ÖS—Östergötlands län, SK—Skåne län, VG—Västra Götalands län, ÖR—Örebro län, SÖ—Södermanlands län, ST—Stockholms län, VL—Värmlands län, DL—Dalarnas län, UP—Uppsala län, VM—Västmanlands län, GV—Gävleborgs län, JM—Jämtlands län, VN—Västernorrlands län, NR—Norrbottens län, VB—Västerbottens län

The principal component analyses (PCA) was undertaken using EIGENSOFT v.6.0.1 (Patterson et al., 2006). The analyses were performed with pseudo‐haploid genomes and excluding of transition sites. To obtain information on genetic affinities between the Birka individual and the modern populations, we performed *f3*‐outgroup statistics using qp3Pop v. 204 (Patterson et al., 2012) and *D* statistics which were calculated using qpDstat of ADMIXTOOLS (Durand, Patterson, Reich, & Slatkin, 2011; Patterson et al., 2012). The results are summarized in Figure 4 and Supporting Information Figure S4.2a‐b, S4.3, S4.4, Table S4.4, and Supplementary Excel Table.

### 2.7 Strontium isotope analyses

Three molar teeth from the lower jaw of individual Bj 581 were submitted to Sr analyses. For comparison teeth of additional five individuals from Birka were also analyzed, Supporting Information Table S5.1. For three individuals, including the warrior woman, all molars (M1–M3) could be studied and for another three, only the first and second molars (M1–M2) were analyzed. Comparative Sr isotopic data in tooth enamel from ten other individuals from Birka as well as mean values of individuals from other parts of Eastern Middle Sweden were collected from earlier publications (Eriksson et al., 2016; Price, Frei, & Naumann, 2015). In order to relate the strontium values to various regions, the local bioavailable strontium isotope (^87^Sr/^86^Sr) baseline should be established as a reference for comparisons (Bentley, 2006; Linderholm, Hedenstierna‐Jonson, Svensk, & Lidén, 2008; Slovak & Paytan, 2011). Unfortunately, baseline data of the Sr isotopic range in the Lake Mälaren Valley is not known. The bedrock in large parts of Eastern Middle Sweden, including Birka, belongs to the Baltic Shield and comprise of Precambrian crystalline (granitic) rocks but also younger metamorphosed rocks, such as sandstone and quartzite (Lindström, qvist, Lundqvist, Calner, & Sivhed, 2011). These rocks may be expected to have different Sr isotopic values. Available data from other regions in Sweden and also the comparative data of humans from Eastern Middle Sweden aid interpretations and are considered sufficient for the purpose at hand.

## 3 RESULTS

Genome‐wide sequence data was generated in order to confirm the biological sex, to support skeletal integrity and also to investigate the genetic relationship of the individual to ancient individuals and modern day groups (S4). We investigated two samples from grave Bj 581, the left canine and the left humerus, which both yielded sufficient amounts of DNA for further analyses (Supporting Information Table S4.1). The DNA was extracted following previously published procedures (Günther et al., 2015). The bone extract contained 0.54% endogenous human DNA and the tooth extract contained 3.88%. The obtained DNA sequences showed all the characteristics of authentic and ancient DNA (Briggs et al., 2007) (Supporting Information Figure S4.1a,b), with mitochondrial contamination estimated to 0.42% (Green et al., 2008).

The Birka warrior was sequenced to mean 0.09× nuclear and 326.5× mitochondrial genome coverage. The mt‐haplogroup was assigned to T2b (Vianello et al., 2013). The total of 11312749 reads mapped to the human genome. When corrected for clonality, the number of reads mapping to X and Y chromosomes were 248,170 and 247, respectively, resulting in the proportion of the alignments (*R*_Y_) equal to 0.001 (*SE* = 0.0001). The cut‐off value for identification of females is *R*_Y_ ≤ 0.016, showing that Bj 581 was a female (Skoglund et al., 2013) (Figure 3). Hence the individual in grave Bj 581 is the first confirmed female high‐ranking Viking warrior. Finally, we note that both biological sex and mtDNA analyses support the single‐individual origin of the analyzed cranial and postcranial remains (same sex and mt‐haplogroup T) (Supporting Information Table S4.3).

The Viking warrior female showed genetic affinity to present‐day inhabitants of the British Islands (England and Scotland), the North Atlantic Islands (Iceland and the Orkneys), Scandinavia (Denmark and Norway) and to lesser extent Eastern Baltic Europe (Lithuania and Latvia) (Figure 4a,b and Supporting Information Figure S4.2a,b). Furthermore, the woman is significantly more similar to these modern northern Europeans than to southern Europeans (Supporting Information Table S4.5). All of those geographical locations are situated within the Viking World. A detailed comparison with modern‐day Swedish individuals from across the entire country shows genetic affinities between the female warrior and southern and south‐central Swedes (Figure 4c).

The strontium isotope values (^87^Sr/^86^Sr) in three teeth (first molar 0.71842, second molar 0.71623, and third molar 0.71687) could suggest mobility in her early years (between the formation age of M1 and M2) (S5 and Supporting Information Figure S5.1a). The Sr values fall in the lower range of other Birka individuals (the mean for all measurements in the present study is 0.7214, sd 0.0048) (Supporting Information Table S5.1 and Figure S5.1a). The Sr ratio falls outside of the local baseline, as estimated on the basis of faunal proxies (Bäckström & Price, 2016; Oras et al., 2016; Price et al., 2015; Price, Peets, Allmäe, Maldre, & Oras, 2016; Peschel, Carlsson, Bethard, & Beaudry, 2017; Wilhelmson & Ahlström, 2015) (Supporting Information Figure S5.1b and S5.2). Thus, the female warrior, was probably of nonlocal origin and, had moved to Birka.

## 4 DISCUSSION

Birka embodies the conceptions of the Viking Age as a period of long distance connections, trade, crafts and warfare. The archaeological material provides a reference for the Viking Age. At Birka, grave Bj 581 was brought forward as an example of an elaborate high‐status male warrior grave. This image of the male warrior in a patriarchal society was reinforced by research traditions and contemporary preconceptions (Moen, 2011). Hence, the biological sex of the individual was taken for granted.

A first osteological analysis done in the 1970ies identified the skeleton as a female, but this could not generate further discussion as the skeleton could not securely be associated to a context. When the sex identification and a proper contextualisation was made, and set in relation to the objects (Kjellström, 2016), questions were still raised if the martial objects in the grave mirrored the identity of the deceased. Similar associations of women buried with weapons have been dismissed, arguing that the armaments could have been heirlooms, carriers of symbolic meaning or grave goods reflecting the status and role of the family rather than the individual (Gardeła, 2013). Male individuals in burials with a similar material record are not questioned in the same way. Furthermore, an argument can be put forward that the grave originally may have held a second, now missing, individual. In which case, the weaponry could have been a part of that individual's grave furnishings, while the remaining female was buried without any objects. However, the distribution of the grave goods within the grave, their spatial relation to the female individual and the total lack of any typically female attributed grave artefacts disputes this possibility.

Do weapons necessarily determine a warrior? The interpretation of grave goods is not straight forward, but it must be stressed that the interpretation should be made in a similar manner regardless of the biological sex of the interred individual. Furthermore, the exclusive grave goods and two horses are worthy of an individual with responsibilities concerning strategy and battle tactics. The skeletal remains in grave Bj 581 did not exhibit signs of antemortem or perimortem trauma which could support the notion that the individual had been a warrior. However, contrary to what could be expected, weapon related wounds (and trauma in general) are not common in the inhumation burials at Birka (e.g., 2 out of 49 confirmed males showed signs of sharp force trauma). A similarly low frequency is noted at contemporaneous cemeteries in Scandinavia (e.g., Helgesson Arcini, 1996). Traces of violent trauma are more common in Viking Age mass burials (e.g., Loe, Boyle, Webb, & Score, 2014; Price et al., 2016).

Although not possible to rule out, previous arguments have likely neglected intersectional perspectives where the social status of the individual was considered of greater importance than biological sex. This type of reasoning takes away the agency of the buried female. As long as the sex is male, the weaponry in the grave not only belong to the interred but also reflects his status as warrior, whereas a female sex has raised doubts, not only regarding her ascribed role but also in her association to the grave goods.

Grave Bj 581 is one of three known examples where the individual has been treated in accordance with prevailing warrior ideals lacking all associations with the female gender (Jesch, 2009) (S1, S2, and S3). Furthermore, the exclusive grave goods and two horses are worthy of an individual with responsibilities concerning strategy and battle tactics. Our results caution against sweeping interpretations based on archaeological contexts and preconceptions. They provide a new understanding of the Viking society, the social constructions and also norms in the Viking Age. The genetic and strontium data also show that the female warrior was mobile, a pattern that is implied in the historical sources, especially when it comes to the extended households of the elite (cf. Steinsland, Sigurđsson, Rekdal, & Beuermann, 2011). The female Viking warrior was part of a society that dominated 8th to 10th century northern Europe. Our results—that the high‐status grave Bj 581 on Birka was the burial of a high ranking female Viking warrior—suggest that women, indeed, were able to be full members of male dominated spheres. Questions of biological sex, gender and social roles are complex and were so also in the Viking Age. This study shows how the combination of ancient genomics, isotope analyses and archaeology can contribute to the rewriting of our understanding of social organization concerning gender, mobility and occupation patterns in past societies.

*Then the high‐born lady saw them play the wounding game*,

*she resolved on a hard course and flung off her cloak;*

*she took a naked sword and fought for her kinsmen's lives*,

*she was handy at fighting, wherever she aimed her blows*.

The Greenlandic Poem of Atli (st. 49) (Larrington, 1996)

## Supporting information

Additional Supporting Information may be found online in the supporting information tab for this article.

Supporting Information 1

Supporting Information 2
