# Supplementary material for: A female Viking warrior confirmed by genomics
Source: Am J Phys Anthropol. 2017 Sep 8;164(4):853–60. doi: 10.1002/ajpa.23308 (PMC5724682; doi:10.1002/ajpa.23308)
Supplement: Supplementary file 2 — Supporting Information 2 [file AJPA-164-853-s002.docx]

# SUPPORTING INFORMATION

# S1 Archaeological context

Birka was the first Viking Age town in Eastern Scandinavia and constitutes one of the most well-known archaeological sites in Northern Europe. Situated on the small island of Björkö in Lake Mälaren, now in the backwaters of Stockholm, it was once on the crossroads of communication in Eastern Central Sweden. Established in the mid-8^th^ century, Birka quickly developed into a major centre of crafts and trade, both in the surrounding region and beyond. Throughout the following century (750 – 860 CE), Birka was part of a social and economic network centred round the coastline of the Baltic that included other urban settlements like Dorestad in the Netherlands, Ribe in Denmark, Haithabu in Germany and Truso in Poland (Sindbæk, 2007).

In the latter part of the 9^th^ century, the significance of these connections declined as Birka became an important node in the Eastern trade network sharing close contacts with similar sites in Ancient Rus’, with Byzantine metropolis Constantinople and even people of the Eurasian steppes. Specialised workshops produced jewellery, textiles and other valuables, that together with slaves were possible to trade for silver and prestigious and exotic goods (Ambrosiani, 2013; Androshchuk, 2013; Hedenstierna-Jonson, 2014).

The inhabitants of the town were buried in extensive cemeteries encircling the urban area. Approximately 3000 graves have been identified within these cemeteries, and a little more than 1100 have been archaeologically examined. Most of the excavations took place in the late 19^th^ century and the results were published in the 1940s (Arbman, 1941). Diverse mortuary practices reflect a heterogeneous population and include cremations following the regional tradition but also various forms of inhumation (Gräslund, 1980). The graves exhibit a great diversity in external and internal construction as well as in grave goods and handling of the body. This diversity reflects not only temporal trends but also a variation in socio-cultural backgrounds of the deceased. There are also differences in the spatial distribution within and between the burial grounds (Gräslund, 1980; Kalmring, 2012). The outer regions of the largest grave field, Hemlanden, and the burial grounds, South of Borg, contain cremations. The inhumations, including the chamber-graves, were situated in close proximity to the settlement-area, inside or just outside the town rampart and in the topographically distinguished burial ground North of Borg. They can be considered to represent the urban population and its changes over time.

Grave Bj 581 (Swedish History Museum, inventory number SHM 34000: Bj 581) was situated in a cemetery north of the hill-fort. The burials, often in coffins and sometimes in chambers, were placed on a natural terrace just outside the main entrance of the fortified area. A space empty of graves leading up to the gate and down to the garrison, marked a lane that connected these. The grave was placed at the very edge of the burial ground overlooking the water (Arbman, 1941; Gräslund, 1980). The fact that the chamber-grave was placed in a vantage point overseeing Birka’s dense center and the Viking Age royal manor at Adelsö indicates the prominence of the buried individual. Furthermore, other burials with extensive grave goods are located in the vicinity. In direct proximity to grave Bj 581 there were graves of men, women and children, all of which were richly furnished. For example, the adjacent male grave (Bj 495) (Arbman, 1941) contained amongst other things a spear, axe, shield and a ringed pin in addition to a fragment of an Arabic coin from the early 900s. In a similar type of liminal position with a landscape viewshed as the female warrior in Bj 581, was yet another weapon burial (Bj 496) (Arbman, 1941); an individual buried with shield, spear, sword, and osteologically-determined as a possible male, 25–30 years of age. The grave goods included stirrups and horse equipment, and although a platform for a horse had been built, no remains of horse bones were found in the chamber, nor were there traces of a gaming set (Figure S1.1a-b). An Arabic silver coin from 900 CE, as well as the objects, indicates that this male warrior was probably contemporary with the female warrior in Bj 581 (Arbman, 1941), but that he presumably was lower in rank.

## ***Other Scandinavian female warriors***

Two Viking Age burials found in southeastern and mid-Norway have been osteologically interpreted as containing young females, but have nevertheless been equipped as if they were warriors. The skeletal remains (Unimus C 22541) (Unimus, 2016) of a slender and petite woman were found in 1900 in a large burial mound located about 1.5 km east of Åsnes church in Hedmark, Norway (Hernæs, 1984). The woman had been buried, east-west oriented, around 950 CE as a fully equipped warrior, with a double-edged sword, an axe, a spear, five arrowheads, a shield, bridles, fragments of a whetstone and probably a file. At her feet lay a horse. The sword lay along the body's left side, but in reverse direction, so that its point was at the woman's head. The osteological analysis of the skeleton showed the interred to be a young female, approx. 18 – 20 years of age (Holck, 1984). It has been argued that despite her outfit, the slender young woman's hand could never have been large enough to reach around the grip of the sword and thus she would have been unable to handle the sword in real combat (Hernæs, 1984), but this must be open to question.

The second female Viking weapon burial seems to have lacked any marker above ground and was found at Aunvollen, close to Snåsavattnet in Nord-Trøndelag (Unimus T 20248) (Unimus, 2016). The grave goods had a conventionally all-male character and consisted of a sword, nine gaming pieces, a sickle, a whetstone, a pair of scissors, a knife and a comb. The osteological analysis showed that the female was c. 20 years old. She was accompanied by a dog. The sword scabbard was made of willow and lined with pelt, there were traces of textiles and down feathers in the grave as well. By her head (oriented towards the south) lay gaming pieces, a whetstone, a comb, scissors and some nails. The sword and sickle were probably placed by her left hip. At a later date a spear and a bead, as well as several nails or rivets were found at the site (Stenvik, 2005).

# S2 Osteological analyses

The buried individual from Bj581 has been interpreted as being buried seated due to the position of the skeleton shown in the illustration by the excavator, Hjalmar Stolpe, where the individual was laying on the right side with the legs slightly bent. The seated posture was originally suggested by Stolpe (Stolpe, 1889), though ignored by subsequent scholars; we are the first to restore this interpretation (Figure S1.1a).

Osteologist Berit Vilkans noted already in the beginning of the 1970s that the skeletal remains in the grave exhibited female characteristics (Vilkans, 1975). However, due to the fact that some finds and osteological materials from Birka have lost their contextual providence during the years of storage, Vilkans’ observations were never commented upon. Nevertheless, except for the missing cranium, the documented remains correspond with the documentation by Stolpe from 1889 (Stolpe, 1889). For example, among the depicted inhumation graves (chamber graves or coffin burials) few seem to have had complete preserved spines when excavated. During the osteological investigation, of at least 245 analysed uncremated skeletons, only four, except for Bj 581, have a complete vertebral column preserved. All bone elements have at one time been marked with the text “Bj 581” in ink. Furthermore they all share the same character and colour on the surface, signifying that they were part of the same individual.

# S3 Archaeological sex assessment

In archaeology, grave goods are often used when assessing the sex of the interred (Petré, 1984; Rundkvist, 2003). Even when there is no remaining skeletal material, the sex or gender of the individual has been determined based on artefactual evidence, though there is clearly a need for caution in such assumptions (Back Danielsson, 2007). As the bias of artefactual sex assessment is unknown, the need for osteological analyses has been stressed, but also the possibility of genetic sex determination (Conkey and Spector, 1984).

# S4 Genetic analyses

## ***DNA Extraction and sequencing***

All laboratory procedures were carried out at facilities designated solely to ancient DNA (aDNA) analyses, located at the Archaeological Research Laboratory (AFL), Stockholm University. DNA was extracted from 85 and 119 mg of bone powder from the left canine and the left humerus respectively (Table S4.1). The surface of the samples was cleaned with either 1% NaOCl (canine) or it was mechanically removed with sandpaper (humerus). Cleaned samples underwent UV irradiation at 265nm, 1J/cm^2^ on each side. Both samples were powdered using dentistry drills and a Dremel®4000 drill. The extraction procedure consisted of overnight digestion (buffer 0.5M EDTA pH 8, 1M Urea, 100 µg/mL Proteinase K) combined with silica-based spin column purification – MinElute PCR Purification Kit (Qiagen), according to the manufactures protocols (Malmström et al., 2009; Yang et al., 1998). Finally, DNA was eluted in 110 μl EB buffer and stored at -20^o^C. Of each obtained DNA extract, 20 μl were used for preparation of blunt-end Illumina genomic libraries (Meyer and Kircher, 2010). The libraries were inspected on 1% agarose gel (0.5x TBE, stained with GelRed™ x10000, Biotium Inc.) and were then amplified with AmpliTaq® Gold DNA Polymerase (Applied Biosystems™) in six separate PCR reactions with different number of cycles for the bone (x14) and the canine (x16) extracts respectively. The amplified products were pooled, purified with magnetic beads (Agencourt AMPure XP, Beckman Coulter); quantified using DNA High Sensitivity Kit with Agilent 2100 Bioanalyzer Instrument (Agilent Technologies) and shotgun sequenced on two Illumina HiSeq platforms. After the initial screening on 0.1^th^ of a lane on Illumina Next-Generation Sequencing (NGS) HiSeq2500 (High Output mode, PE 2x125bp, validated method) the library made from the tooth extract was further sequenced in total on one Illumina HiSeqX lane (v2.5, PE 2x150bp, validated method). The sequencing was performed at the Science for Life Laboratory Sequencing Centre in Stockholm. Raw DNA data was sorted into individual samples based on tagged sequences (de-multiplexed), quality-controlled and delivered to UPPNEX (UPPmax NEXt Generation sequence Cluster & Storage) (Lampa et al., 2013).

## ***Sequence analyses***

The computations were performed on resources provided by SNIC through Uppsala Multidisciplinary Center for Advanced Computational Science (UPPMAX) under the following projects: b2013240, b2015307 and b2016056. Sequence data was analysed following Günther et al 2015 (Günther et al., 2015) and Omrak et al 2016 (Omrak et al., 2016).

In short, de-multiplexed sequencing reads, either 125bp or 150bp pair-end were merge and trimmed using M. Kircher’s ‘MergeReadsFastQ_cc.py’ script (Kircher, 2012). Obtained sequences were then mapped to the human reference genomes (build 36 and 37) with BWA v. 0.7.13 (Li and Durbin, 2010) (aln with non-default parameters: -l 16500 -n 0.01 -o 2) and PCR duplicates (sequences with identical start-end genome positions) were removed by merging into consensus sequences using FilterUniqueSAMCons.py (Kircher, 2012). Sequences shorter than 35bp or with more than 10% mismatches to the human reference genome were removed. Bam files from different sequencing runs were merged using SAMtools v.0.1.19 (Li et al., 2009).

Obtained DNA fragments were checked for presence of 3’ and 5’ degradation patterns characteristic of ancient DNA(Briggs et al., 2007; Hansen et al., 2001; Hofreiter et al., 2001; Orlando et al., 2011; Sawyer et al., 2012) using PMDtools (Skoglund et al., 2014) (Figure S4.1).

Levels of contamination were estimated by counting consensus and non-consensus reads at private and near-private polymorphic sites in the consensus sequence of our sample (<5% as identified in a reference panel of 311 modern mitochondrial sequences) which allows for identification of multiple templates (Green et al., 2008). The consensus sequence was called with samtools, only using reads and bases with a minimum mapping quality of 30 and a minimum base quality of 30. Sites where the consensus allele was G or C were excluded from the estimation of contamination and only positions with a minimum coverage of 10 were taken into account.

Mitochondrial DNA (mtDNA) was assigned to a specific haplogroup using an online tool, HAPLOFIND (Vianello et al., 2013). The assignments were inspected manually by aligning mitochondrial consensus sequences to the revised Cambridge Reference Sequence (rCRS) (Anderson et al., 1981; Andrews et al., 1999). The consensus listed in table S4.2. The DNA sequences obtained from the humerus also allowed for broad mitochondrial DNA (mtDNA) haplogroup assignment to Hg T*. Even though the assignment could not be further narrowed down due to insufficient mtDNA genome coverage (×0.5) the results are in agreement with the haplogroup assignment obtained from the tooth DNA extract; T2b (Table S4.2). sequences were aligned in BioEdit v.7.2.5 (Hall, 1999), while the NGS contigs were inspected in the Integrative Genomics Viewer (IGV) 2.3.32 (Robinson et al., 2011; Thorvaldsdóttir et al., 2013). Observed mutations were checked against PhyloTree – mtDNA tree build 17 (18^th^ February 2016) (van Oven and Kayser, 2009) and are reported in Table S4.1.

## ***Biological sex assignment***

The biological sex of the individual was estimated based one the ratio of sequences aligning to the two sex chromosomes, X and Y (Skoglund et al., 2013). While female individuals are carriers of two X chromosomes, males carry one X and one Y chromosome. Note that the X chromosome contains a small region that is homologous with a small region on the Y chromosome (also known as the XY region), and a small fraction of sequences can be mapped equally well to the X chromosome and the Y chromosome (Jobling et al., 2004).Thus by assessing the ratio between sequence alignments to the two chromosomes it is possible to establish the biological sex of the individual. The method utilizes the following formulae, *R*_Y_ = *n*_Y_/(*n*_X_ + *n*_Y_), where *n*_X_ is the number of alignments to chromosome X, *n*_Y_ is the number of alignments to chromosome Y, and *R*_Y_ represents the ratio between alignments *n*_Y_ and the sum of all alignments to both sex chromosomes. Females are identified when *R*_Y_ ≤ 0.016 and males when *R*_Y_ ≥ 0.077 (Skoglund et al., 2013). Additionally, a 95% confidence interval (CI) is computed by approximation as *R*_Y_ ± 1.96 × *R*_Y_ × (1- *R*_Y_)/(*n*_X_ + *n*_Y_). We performed the biological sex assignment on the two extracts from the Birka warrior as well as on seven previously published ancient genomes of known biological sex. The Birka warrior bone extract yielded an *R*_Y_ value of (*R*_Y_ = 0.002) and the tooth extract (*R*_Y_ = 0.001), both well within female range *R*_Y_ ≤ 0. 016^72^. The obtained values are presented in Table S4.3 and main text Figure 3.

## ***Reference population panel***

The genetic data from the Birka warrior was merged with three different population reference data-sets consisting of genotype SNP data from: the Human Origins dataset (Lazaridis et al., 2014; Patterson et al., 2012), The Population Reference Sample – POPRES (Nelson et al., 2008), and the Swedish reference (Salmela et al., 2011). At each SNP site, a random read with minimum mapping quality 30 and minimum base quality 30 at the SNP site was picked from the Birka warrior and assumed to be in homozygous state for the individual. Additionally, in order to allow for population comparisons between different data-sets with the same outgroup population, both the POPRES data and the Swedish reference panel were merged with low-coverage Yoruban sequence data by randomly sampling one read from each of 60 individuals from the pilot phase of the 1000 Genomes Project (The 1000 Genomes Project Consortium, 2012). The merged datasets were used to perform: Principal Component Analyses to visualise the samples position within modern day genetic variation; outgroup *f*_3_-statistic to assess the amount of shared genetic drift between our investigated individual and the reference populations; *D*-statistics to trace direction of the observed gene flow.

## ***Population comparisons***

The principal component analyses (PCA) was performed using the Human Origins data-set consisting of 21 European populations (Lazaridis et al., 2014; Patterson et al., 2012), the POPRES data-set consisting of 37 populations (Nelson et al., 2008) (Figure S4.2a-b) and the Swedish reference (Figure S4.3) (Salmela et al., 2011). The analyses were undertaken using EIGENSOFT v.6.0.1 (Patterson et al., 2006). The PCA was performed excluding transitions and randomly picking one allele if two alleles were available for a given locus, thus creating pseudo-haploid genomes both for the modern reference and the ancient sample.

To obtain information on individual’s relatedness to the modern populations, we performed *f3*-outgroup statistics using qp3Pop v. 204 of the form (O; A, B) (Patterson et al., 2012). The outgroup (O) is expected equidistant to both the tested sample (A) and any of the other European or Swedish populations from the reference panels (B). Thus the result of outgroup *f3*-statistics (assuming no gene-flow between the test populations and the outgroup) reflects the amount of genetic affinity shared between test populations, A and B. The analyses were performed with all three data-sets (Human Origins and YRI+POPRES, and Swedish DB + YRI using 10401, 3104 and 5421 SNPs respectively). The outgroup (O) was the Yoruban population, the Birka warrior as the test individual A, and any of the populations from the reference panel as the test population B. The analyses were performed with 648, 456 and 485 jackknife blocks. The results are presented in Figure 4, Figure S4.4 and detailed information can be found in Supplementary Data Excel sheet.

Finally, to test for deviations from tree-like population history between the samples, we used *D*-statistics which were calculated using qpDstat of ADMIXTOOLS (Durand et al., 2011; Patterson et al., 2012). Deviations from tree-like topology can be interpreted as evidence of gene-flow between populations pointing to closer affinity among pairs of tested samples. The standard errors were estimated by performing block jackknife over blocks of the genome (Durand et al., 2011). Results of all tests performed are listed in the Supplementary Excel Table (SupplementaryData.xls). Summarised results testing whether the Viking warrior female was closer to the western or eastern populations (the British Islanders or the inhabitants of the Baltic States) were reported in Table S4.4. The individual was found to share affinities (*D*-statistics, |Z| > 2) with some Eastern groups, especially Estonians and Latvians as well as with western North Atlantic Islanders (English from Kent, Icelanders and the Scots) (Table S4.4). We also provide statistical evidence that the individual shows greater affinity to the northern and western populations listed in Table S4.4. than other southern European example populations from outside the Viking world, Table S4.5. The tests were calculated as: D (outgroup, Birka warrior; non listed population, listed population). The positive values of D support greater affinity of the Birka warrior to populations listed in Table S4.4 than a selection of non-listed populations.

# S5 Strontium Isotope Analyses

In addition to the genetic analyses, samples of teeth of the individual in grave Bj 581 were submitted for Sr analyses to Curt-Engelhorn-Zentrum Archäometrie gGmbH, Mannheim (PhD Corina Knipper) with the aim to highlight questions on mobility and movement at Birka. Analyses of stable isotopes of sulphur (δ^34^S) have previously shown that the population at Birka had a heterogeneous geographic origin and also that there was a variation in dietary patterns (based on δ^13^C and δ^15^N) (Linderholm et al., 2008)^.^

Strontium signatures (^87^Sr/^86^Sr) in human tooth enamel may be used to reconstruct mobility patterns and to distinguish between local and non-local individuals (Alexander Bentley, 2006; Linderholm et al., 2008; Slovak and Paytan, 2011). Strontium replaces calcium in the formation of the skeleton and is incorporated into the enamel through the food chain, i.e. the consumed (local) food and water, at the time of tooth crown formation and mineralization. Thus, the composition of different isotopes in the enamel reflects (mainly) the bedrock signature in a given region, and variation between different teeth in an individual indicates mobility or movement during childhood and adolescence. There is no or very limited diagenetic alteration. The analyses were performed at Curt-Engelhorn-Zentrum Archäometrie gGmbH following standard procedures. The principals of the analytic method for Sr analyses have been described in several publications (Alexander Bentley, 2006; Price et al., 2002; Slovak and Paytan, 2011). The analyses targeted the composition of Sr isotopes ^87^Sr and ^86^Sr in the enamel of molar teeth. There is a large variation in the timing of tooth crown mineralization and broad estimates have to be adopted. The crowns of the permanent molars are mineralized broadly from c.0.5m in utero to c.3 yrs for M1, 2-3 yrs to c.8 yrs for M2 and finally 7-16 yrs for M3 (Hillson, 1986; Schour and Massler, 1941; White, 2005).

Our results show a variation in Sr isotope values between 0.7122 and 0.7267 (Table S5.1 and Figure S5.1a). The mean values for all measurements is 0.7214 (sd 0.0048), for M1 0.7213 (sd 0.0046), for M2 0.7220 (sd 0.0057) and for M3 0.7206 (sd 0.0027). The variation between different teeth of the individuals is limited. Four individuals exhibit values above 0.7220 for all teeth, while two individuals exhibit lower values, one of them being the warrior woman in grave Bj 581. Sr values are available from several regions of Eastern Middle Sweden all showing high ratios above 0.720. Published data of Sr isotopic values of ten Birka individuals exhibit a lower mean value but a wider range (Price et al., 2015). These values vary between 0.7103 and 0.7335, with a mean of 0.7174 sd 0.0074) (but which burials and which teeth were analyzed are not reported). Mean Sr values in human teeth from Central Sweden vary between 0.7151 (Stockholm) and 0.7321 (Björklinge) which indirectly show that the local bedrock exhibit high Sr isotope values (Price et al., 2015). However, these samples may include non-local individuals. The reported ranges are in agreement with a recent study that reported a strontium isotopic range ^87^Sr/^86^Sr from 0.731 to 0.743 for soil samples taken in similar geological environments as around Birka in Närke and Sörmland (Eriksson et al., 2016). Strontium isotope ratios of faunal samples from other regions of the Baltic exhibit lower values than Eastern Middle Sweden (Figure S5.2.). This, it looks like two of the studied individuals here were non-locals and one of them was the female warrior. Apparently most of the individuals from Birka previously analyzed were, in fact, non-locals.

Our data deviates from the previous study in Sr isotopic values of Birka individuals. The earlier published dataset is characterized by low and high values, while our data is more homogeneous and falls within the lower range of the variation (Table S5.2 and Figure S5.1b). The differences are probably due to the character of the site: we may expect that Birka attracted people from a vast geographical region. The wide range in Sr values demonstrates the heterogeneous character of the population in Birka. It is of interest that the individuals buried in inhumation graves in Birka have not adopted the local tradition of cremation, although this was a common burial practice also in Birka. Additionally we need to consider that the town was established without an association to any older type of settlement on the site (Holmquist, 1993; Linderholm et al., 2008). There is ample archaeological evidence that people came to Birka and settled there, both from the surrounding region but also from further away.

# REFERENCES

Ambrosiani, B. (2013). *Birka 1680-2013: ett långvarigt forskningsprojekt = a long-term research project*. Stockholm: Paniba HB.

Anderson, S., Bankier, A. T., Barrell, B. G., de Bruijn, M. H., Coulson, A. R., Drouin, J., Eperon, I. C., Nierlich, D. P., Roe, B. A., Sanger, F., Schreier, P. H., Smith, A. J., Staden, R., Young, I. G.. (1981). Sequence and organization of the human mitochondrial genome. *Nature* 290, 457–465.

Andrews, R. M., Kubacka, I,, Chinnery, P. F., Lightowlers, R. N., Turnbull, D. M., Howell, N. (1999). Reanalysis and revision of the Cambridge reference sequence for human mitochondrial DNA. *Nature Genetics* 23, 147.

Androshchuk, F. (2013). *Vikings in the East: Essays on Contacts along the Road to Byzantium (800-1100). (Studia Byzantina Upsaliensia)*. Uppsala: Acta Universitatis Upsaliensis.

Arbman, H. (1941). *Birka. Untersuchungen und Studien I. Die Gräber. Text und Tafeln*. Stockholm: Kungl. Vitterhets-, historie- och antikvitetsakademien.

Back Danielsson, I-M. (2007). *Masking moments : the transitions of bodies and beings in Late Iron Age Scandinavia*. Stockholm, Department of Archaeology and Classical Studies, Stockholm University.

Bentley, R. A. (2006). Strontium Isotopes from the Earth to the Archaeological Skeleton: A Review. *Journal of Archaeological Method and Theory* 13, 135–187.

Briggs, A. W., Stenzel, U., Johnson, P. L. F., Green, R. E., Kelso, J., Prüfer, K., Meyer, M., Krause, J., Ronan, M. T., Lachmann, M., Pääbo, S. (2007). Patterns of damage in genomic DNA sequences from a Neandertal. *Proceedings of National Academy of Science U S A* 104, 14616–14621.

Bäckström, Y. & Price, D. (2016). Social identity and mobility at a preindustrial mining complex, Sweden. *Journal of Archaeological Science* 66:154–168.

Conkey, M. W., Spector, J. D. (1984). Archaeology and the Study of Gender. *Advances in Archaeological Method and Theory* 7, 1-38.

Durand, E. Y., Patterson, N., Reich, D., Slatkin, M. (2011). Testing for ancient admixture between closely related populations. *Molecular Biology and Evolution* 28, 2239–2252.

Eriksson, G., Frei, K. M., Howcroft, R., Gummesson, S., Molin, F., Lidén, K., Frei, R., Hallgren, F. (2016). Diet and mobility among Mesolithic hunter-gatherers in Motala (Sweden) - The isotope perspective. *Journal of Archaeological Science: Reports* Available at: <http://www.sciencedirect.com/science/article/pii/S2352409X16302097>

Gräslund, A-S. (1980). *Birka : Untersuchungen und Studien IV, The burial customs : a study of the graves on Björkö*. Stockholm, Almqvist & Wiksell international.

Green, R. E., Malaspinas, A. S., Krause, J., Briggs, A. W., Johnson, P. L., Uhler, C., Meyer, M., Good, J. M., Maricic, T., Stenzel, U., Prüfer, K., Siebauer, M., Burbano, H. A., Ronan, M., Rothberg, J. M., Egholm, M., Rudan, P., Brajković, D., Kućan, Z., Gušić, I., Wikström, M., Laakkonen, L., Kelso, J., Slatkin, M., Pääbo, S. (2008). A complete Neandertal mitochondrial genome sequence determined by high-throughput sequencing. *Cell* 134, 416–426.

Günther, T., Valdiosera, C., Malmström, H., Ureña, I., Rodriguez-Varela, R., Sverrisdóttir, Ó. O., Daskalaki, E. A., Skoglund, P., Naidoo, T., Svensson, E. M., Bermúdez de Castro, J. M., Carbonell, E., Dunn, M., Storå, J., Iriarte, E., Arsuaga, J. L., Carretero, J-M., Götherström, A., Jakobsson, M. (2015). Ancient genomes link early farmers from Atapuerca in Spain to modern-day Basques. *Proceedings of National Academy of Science* 112, 11917–11922.

Hall, T. A. (1999). BioEdit:a user-friendly biological sequence alignment editor and analysis program for Windows 95/98/NT. *Nucleic Acids Symposium Series* 41, 95–98.

Hansen, A., Willerslev, E., Wiuf, C., Mourier, T., Arctander, P. (2001). Statistical evidence for miscoding lesions in ancient DNA templates. *Molecular Biology and Evolution* 18, 262–5.

Hedenstierna-Jonson, C. (2014). She came from another place, on the burial of a young girl in Birka (Bj463). In M. Hem Erikse, U. Pedersen, B. Runderget, I. Axelsen, H. Lund Berg (Eds), *Viking worlds: Things, spaces and movement* (pp 90–101). Oxford, Oxbow Books.

Hernæs, P. (1984). C 22541 a-g. Et gammelt funn tolkes på ny. *Nicolay* 43, 31–39.

Hillson, S. (1986). *Teeth*. Cambridge, Cambridge Univ. Press.

Hofreiter, M., Jaenicke, V., Serre, D., von Haeseler, A., Pääbo, S. (2001). DNA sequences from multiple amplifications reveal artifacts induced by cytosine deamination in ancient DNA. *Nucleic Acids Research* 29, 4793–4799.

Holck, P. (1984). Antropologisk kommentar. In P. Hernæs, C 22541 a-g. Et gammelt funn tolkes på ny. *Nicolay* 43, 37–38.

Holmquist, L. 1993. *Aspects on Birka. Investigations and surveys 1976-1989*. Stockholm, Department of Archaeology and Classical Studies, Stockholm University.

Jobling, M. A., Hurles, M., Tyler-Smith, C. (2004). *Human evolutionary genetics: origins, peoples & disease*. New York, Garland Science.

Kalmring, S. (2012). The Birka Proto-Town GIS - A Source for Comprehensive Studies of Björkö. *Fornvännen* 107, 253.

Kircher, M. (2012). Analysis of High-Throughput Ancient DNA Sequencing Data. *Methods in Molecular Biology* 840, 197-228.

Lampa, S., Dahlo, M., Olason, P., Hagberg, J., Spjuth, O. (2013). Lessons learned from implementing a national infrastructure in Sweden for storage and analysis of next-generation sequencing data. *Gigascience* 2, 9..

Lazaridis, I., Patterson, N., Mittnik, A., Renaud, G., Mallick, S., Kirsanow, K., Sudmant, P. H., Schraiber, J. G., Castellano, S., Lipson, M., Berger, B., Economou, C., Bollongino, R., Fu, Q., Bos, K. I., Nordenfelt, S., Li, H., de Filippo. C., Prufer, K., Sawyer, S., Posth, C., Haak, W., Hallgren, F., Fornander, E., Rohland, N., Delsate, D., Francken, M., Guinet, J-M., Wahl, J., Ayodo, G., Babiker, H. A., Bailliet, G., Balanovska, E., Balanovsky, O., Barrantes, R., Bedoya, G., Ben-Ami, H., Bene, J., Berrada, F., Bravi, C. M., Brisighelli, F., Busby, G. B. J., Cali, F., Churnosov, M., Cole, D. E. C., Corach, D., Damba, L., van Driem, G., Dryomov, S., Dugoujon, J-M., Fedorova, S. A., Gallego Romero, I., Gubina, M., Hammer, M., Henn, B. M., Hervig, T., Hodoglugil, U., Jha, A. R., Karachanak-Yankova, S., Khusainova, R., Khusnutdinova, E., Kittles, R., Kivisild, T., Klitz, W., Kucinskas, V., Kushniarevich, A., Laredj, L., Litvinov, S., Loukidis, T., Mahley, R. W., Melegh, B., Metspalu, E., Molina, J., Mountain, J., Nakkalajarvi, K., Nesheva, D., Nyambo, T., Osipova, L., Parik, J., Platonov, F., Posukh, O., Romano, V., Rothhammer, F., Rudan, I., Ruizbakiev, R., Sahakyan, H., Sajantila, A., Salas, A., Starikovskaya, E. B., Tarekegn, A., Toncheva, D., Turdikulova, S., Uktveryte, I., Utevska, O., Vasquez, R., Villena, M., Voevoda, M., Winkler, C. A, et al. (2014). Ancient human genomes suggest three ancestral populations for present-day Europeans. *Nature* 513, 409–413.

Li, H., Durbin, R. (2010). Fast and accurate long-read alignment with Burrows–Wheeler transform. *Bioinformatics* 26, 589–595.

Li, H., Handsaker, B., Wysoker, A., Fennell, T., Ruan, J., Homer, N., Marth, G., Abecasis, G., Durbin, R., Subgroup 1000 Genome Project Data Processing. (2009). The Sequence Alignment/Map format and SAMtools. *Bioinformatics* 25, 2078–2079.

Linderholm, A., Hedenstierna-Jonson, C., Svensk, O., Lidén, K. (2008). Diet and status in Birka: stable isotopes and grave goods compared. *Antiquity* 82, 446–461.

Malmström, H., Gilbert, M. T., Thomas, M. G., Brandström, M., Storå, J., Molnar, P., Andersen, P. K., Bendixen, C., Holmlund, G., Götherström, A., Willerslev, E. (2009). Ancient DNA Reveals Lack of Continuity between Neolithic Hunter-Gatherers and Contemporary Scandinavians. *Current Biology* 19, 1758–1762.

Meyer, M., Kircher, M. (2010). Illumina sequencing library preparation for highly multiplexed target capture and sequencing. *Cold Spring Harbor Protocols* 2010:pdb.prot5448.

Nelson, M. R., Bryc, K., King, K. S., Indap, A., Boyko, A. R., Novembre, J., Briley, L. P., Maruyama, Y., Waterworth, D. M., Waeber, G., Vollenweider, P., Oksenberg, J. R., Hauser, S. L., Stirnadel, H. A., Kooner, J. S., Chambers, J. C., Jones, B., Mooser, V., Bustamante, C. D., Roses, A. D., Burns, D. K., Ehm, M. G., Lai, E. H. (2008). The Population Reference Sample, POPRES: a resource for population, disease, and pharmacological genetics research. *American Journal of Human Genetics* 83, 347–358.

Omrak, A., Günther, T., Valdiosera, C., Svensson, E. M., Malmström, H., Kiesewetter, H., Aylward, W., Storå, J., Jakobsson, M., Götherström, A. (2016). Genomic Evidence Establishes Anatolia as the Source of the European Neolithic Gene Pool. *Current Biology* 26, 270–275.

Oras E., Lang V., Rannamäe E., Varul L., Konsa M., Limbo-Simovart J., Vedru G., Laneman M., Malve M. & Price D. T. (2016). Tracing prehistoric migration: isotope analysis of Bronze and Pre-Roman Iron Age coastal burials in Estonia. *Estonian Journal of Archaeology* 20: 3–32.

Orlando, L., Ginolhac, A., Raghavan, M., Vilstrup, J., Rasmussen, M., Magnussen, K., Steinmann, K. E., Kapranov, P., Thompson, J. F., Zazula, G., Froese, D., Moltke, I., Shapiro, B., Hofreiter, M., Al-Rasheid, K. A., Gilbert, M. T., Willerslev, E. (2011). True single-molecule DNA sequencing of a pleistocene horse bone. *Genome Research* 21, 1705–1719.

van Oven, M., Kayser, M. (2009). Updated comprehensive phylogenetic tree of global human mitochondrial DNA variation. *Human Mutatation* 30, E386-94.

Patterson, N., Pricem A. L., Reich, D. (2006). Population Structure and Eigenanalysis. *PLoS Genetics* 2:e190.

Patterson, N., Moorjani, P., Luo, Y., Mallick, S., Rohland, N., Zhan, Y., Genschoreck, T., Webster, T., Reich, D. (2012). Ancient admixture in human history. *Genetics* 192, 1065–1093.

Peschel E. P., Carlsson D., Bethard J., Beaudry M. C. 2017. Who resided in Ridanäs?: A study of mobility on a Viking Age trading port in Gotland, Sweden. *Journal of Archaeological Science: Reports*. 13:175-184.

Petré, B. (1984). *Arkeologiska undersökningar på Lovö. D. 2, Fornlämning RAÄ 27, Lunda = [The Iron Age cemetery, RAÄ 27, Lunda] : [the excavations 1971-1978]*. Stockholm, Almqvist & Wiksell International.

Price, T. D., Burton, J. H., Bentley, R. A. (2002). The Characterization of Biologically Available Strontium Isotope Ratios for the Study of Prehistoric Migration. *Archaeometry* 44, 117.

Price, T. D., Frei, K. M., Naumann, E. (2015). Isotopic Baselines in the North Atlantic Region. *Journal of the North Atlantic* 7, 103.

Robinson, J. T., Thorvaldsdottir, H., Winckler, W., Guttman, M,, Lander, E. S., Get,z G., Mesirov, J. P. (2011). Integrative genomics viewer. *Nature Biotechnology* 29, 24–26.

Rundkvist, M. (2003). *Barshalder. 1, A cemetery in Grötlingbo and Fide parishes, Gotland, Sweden, c. AD 1-1100 : excavations and finds 1826-1971*. Stockholm, Department of Archaeology and Classical Studies, Stockholm University..

Salmela, E., Lappalainen, T., Liu, J., Sistonen, P., Andersen, P. M., Schreiber, S., Savontaus, M-L., Czene, K., Lahermo, P., Hall, P., Kere, J. (2011). Swedish population substructure revealed by genome-wide single nucleotide polymorphism data. *PLoS One* 6:e16747.

Sawyer, S., Krause, J., Guschanski, K., Savolainen, V., Pääbo, S. (2012). Temporal Patterns of Nucleotide Misincorporations and DNA Fragmentation in Ancient DNA. *PLoS One* 7:e34131.

Schour, I., Massler, M. (1941). The development of the human dentition. *Journal of American Dental Association* 28, 1153–60.

Sindbæk, S. M. (2007). Networks and nodal points: The emergence of towns in early Viking Age Scandinavia. *Antiquity* 81, 119–132.

Skoglund, P., Storå, J., Götherström, A., Jakobsson, M. (2013). Accurate sex identification of ancient human remains using DNA shotgun sequencing. *Journal of Archaeological Science* 40, 4477–4482.

Skoglund, P., Northoff, B. H., Shunkov, M. V., Derevianko, A. P., Pääbo, S., Krause, J., Jakobsson, M. (2014). Separating endogenous ancient DNA from modern day contamination in a Siberian Neandertal. *Proceedings of National Academy of Science U S A* 111, 2229–34.

Slovak, N. M., Paytan, A. (2011). Applications of Sr Isotopes in Archaeology. In M. Baskaran (Ed.), *Handbook of Environmental Isotope Geochemistry* (pp. 743–768.). Berlin Heidelberg, Springer-Verlag.

Stenvik, L. (2005). Sosiale forskjeller. In I. Bull (Ed.), *Trøndelags historie. Landskapet blir landsdel. Fram til 1350* (pp. 147–157), Trondheim, Tapir forlag.

Stolpe, H. (1889). Ett och annat på Björkö. *Ny Illustrerad Tidning* 25.

The 1000 Genomes Project Consortium. (2015). A global reference for human genetic variation. *Nature* 526, 68–74.

Thorvaldsdóttir, H., Robinson, J. T., Mesirov, J. P. (2013). Integrative Genomics Viewer (IGV): high-performance genomics data visualization and exploration. *Briefings in Bioinformatics* 14, 178–192.

Unimus = museum collections of the universities of Norway (2016). Available from: www.unimus.no/arkeologi/forskning/index.php

Vianello, D., Sevini. F., Castellani, G., Lomartire, L., Capri, M., Franceschi,.C. (2013). HAPLOFIND: A New Method for High-Throughput mtDNA Haplogroup Assignment. *Human Mutatation* 34, 1189–1194.

Vilkans, B. (1975). *101 gravar från Björkö, Adelsö sn, Up. Osteologisk undersökning*. Rapportserie från Osteologiska enheten. Report available at The Osteological unit, The Swedish History Museum.

Wilhelmson H. & Ahlström T. (2015). Iron Age migration on the island of Öland: Apportionment of strontium by means of Bayesian mixing analysis. *Journal of Archaeological Science* 64: 30-45

White, T. D., Folkens, P. A. (2005). *The human bone manual. Amsterdam*: Elsevier Academic.

Yang, D. Y., Eng, B., Waye, J. S., Dudar, J. C., Saunders, SR. (1998). Technical note: improved DNA extraction from ancient bones using silica-based spin columns. *American Journal of Physical Anthropology* 105, 539–43.

**Table S4.1.** Summary statistics for sequence data obtained from the two libraries. Contamination patterns could not be determined in the humerus extract (nd) due to insufficient mtDNA genome coverage (minimum x10).

**Table S4.2.** Mitochondrial genome coverage and polymorphisms recorded against rCRS. *Mutations in bold define haplogroup T2b from macro-haplogroup R0 (mtDNA tree Build 17, 18 Feb 2016) (van Oven and Kayser, 2009).

**Table S4.3.** Summary statistics of sex determination for the Birka warrior and the reference individuals. * canine extract, **humerus extract

**Table S4.4.** Summary D-statistics results. The tests were arranged as D (A,B;X,Y) with outgroup (A), the Birka warrior (B), and comparative populations (X,Y). The DB (database) used in various tests was either HO (Human Origins) (Lazaridis et al., 2014; Patterson et al., 2012) or POPRES (The Population Reference Sample) (Nelson et al., 2008) merged with 60 Yoruban individuals from the 1000 Genomes Project (The 1000 Genome Project Consortium, 2015).

**Table S4.5.** D-statistics results supporting greater affinity of the Birka Warrior to the Northern populations from listed in Table S4.4 than other European example populations from outside the Viking world. The tests were calculated as: D(outgroup, warrior lady; non listed population, listed population). The positive values of D support greater affinity of Birka warrior to populations listed in Table S4.4 than non-listed populations. Most Z scores are statistically significant (*).

| A | B | X | Y | D | Z | SE | Loci | DB |
| --- | --- | --- | --- | --- | --- | --- | --- | --- |
| Yoruba | Birka warrior | Cypriot | EnglishKentGBR | 0.0549 | 5.408* | 0.0102 | 10401 | HO |
| Yoruba | Birka warrior | ItalianTuscan | EnglishKentGBR | 0.0294 | 2.877* | 0.0102 | 10401 | HO |
| Yoruba | Birka warrior | French | EnglishKentGBR | 0.0189 | 2.206* | 0.0086 | 10401 | HO |
| Yoruba | Birka warrior | SpanishAragonIBS | EnglishKentGBR | 0.0346 | 3.159* | 0.0110 | 10401 | HO |
| Yoruba | Birka warrior | Cypriot | Estonian | 0.05 | 5.401* | 0.0093 | 10401 | HO |
| Yoruba | Birka warrior | ItalianTuscan | Estonian | 0.0247 | 2.662* | 0.0093 | 10401 | HO |
| Yoruba | Birka warrior | French | Estonian | 0.0141 | 2.106* | 0.0067 | 10401 | HO |
| Yoruba | Birka warrior | SpanishAragonIBS | Estonian | 0.0299 | 2.973* | 0.0101 | 10401 | HO |
| Yoruba | Birka warrior | Cypriot | Icelandic | 0.0532 | 6.047* | 0.0088 | 10401 | HO |
| Yoruba | Birka warrior | ItalianTuscan | Icelandic | 0.028 | 3.266* | 0.0086 | 10401 | HO |
| Yoruba | Birka warrior | French | Icelandic | 0.0175 | 2.77* | 0.0063 | 10401 | HO |
| Yoruba | Birka warrior | SpanishAragonIBS | Icelandic | 0.0332 | 3.355* | 0.0099 | 10401 | HO |
| YRI | Birka warrior | Cyprus | Latvia | 0.0921 | 2.517* | 0.0366 | 3104 | POPRES+YRI |
| YRI | Birka warrior | Spain | Latvia | 0.0843 | 2.635* | 0.0320 | 3104 | POPRES+YRI |
| YRI | Birka warrior | Italy | Latvia | 0.0829 | 2.557* | 0.0324 | 3104 | POPRES+YRI |
| YRI | Birka warrior | Cyprus | Netherlands | 0.0257 | 1.321 | 0.0195 | 3104 | POPRES+YRI |
| YRI | Birka warrior | Italy | Netherlands | 0.0162 | 1.973 | 0.0082 | 3104 | POPRES+YRI |
| YRI | Birka warrior | Spain | Netherlands | 0.0173 | 2.12* | 0.0082 | 3104 | POPRES+YRI |
| YRI | Birka warrior | Italy | Belgium | 0.0086 | 1.467 | 0.0059 | 3104 | POPRES+YRI |
| YRI | Birka warrior | Cyprus | Belgium | 0.0181 | 1.011 | 0.0179 | 3104 | POPRES+YRI |
| YRI | Birka warrior | Spain | Belgium | 0.0097 | 1.748 | 0.0055 | 3104 | POPRES+YRI |
| YRI | Birka warrior | Italy | Germany | 0.0139 | 2.857* | 0.0049 | 3104 | POPRES+YRI |
| YRI | Birka warrior | Cyprus | Germany | 0.0234 | 1.328 | 0.0176 | 3104 | POPRES+YRI |
| YRI | Birka warrior | Spain | Germany | 0.015 | 3.008* | 0.0050 | 3104 | POPRES+YRI |
| YRI | Birka warrior | Italy | Sweden | 0.0138 | 1.316 | 0.0105 | 3104 | POPRES+YRI |
| YRI | Birka warrior | Cyprus | Sweden | 0.0234 | 1.177 | 0.0199 | 3104 | POPRES+YRI |
| YRI | Birka warrior | Spain | Sweden | 0.0149 | 1.424 | 0.0105 | 3104 | POPRES+YRI |

**Table S5.1.** Strontium values analyzed from six individuals from Birka
(Analysis by C. Knipper, Mannheim).

**Table S5.2.** The distribution of strontium isotopic values (^87^Sr/^86^Sr) in teeth of Birka individuals. Data in Table S5.1 and Price et al. (Price et al., 2015).

**
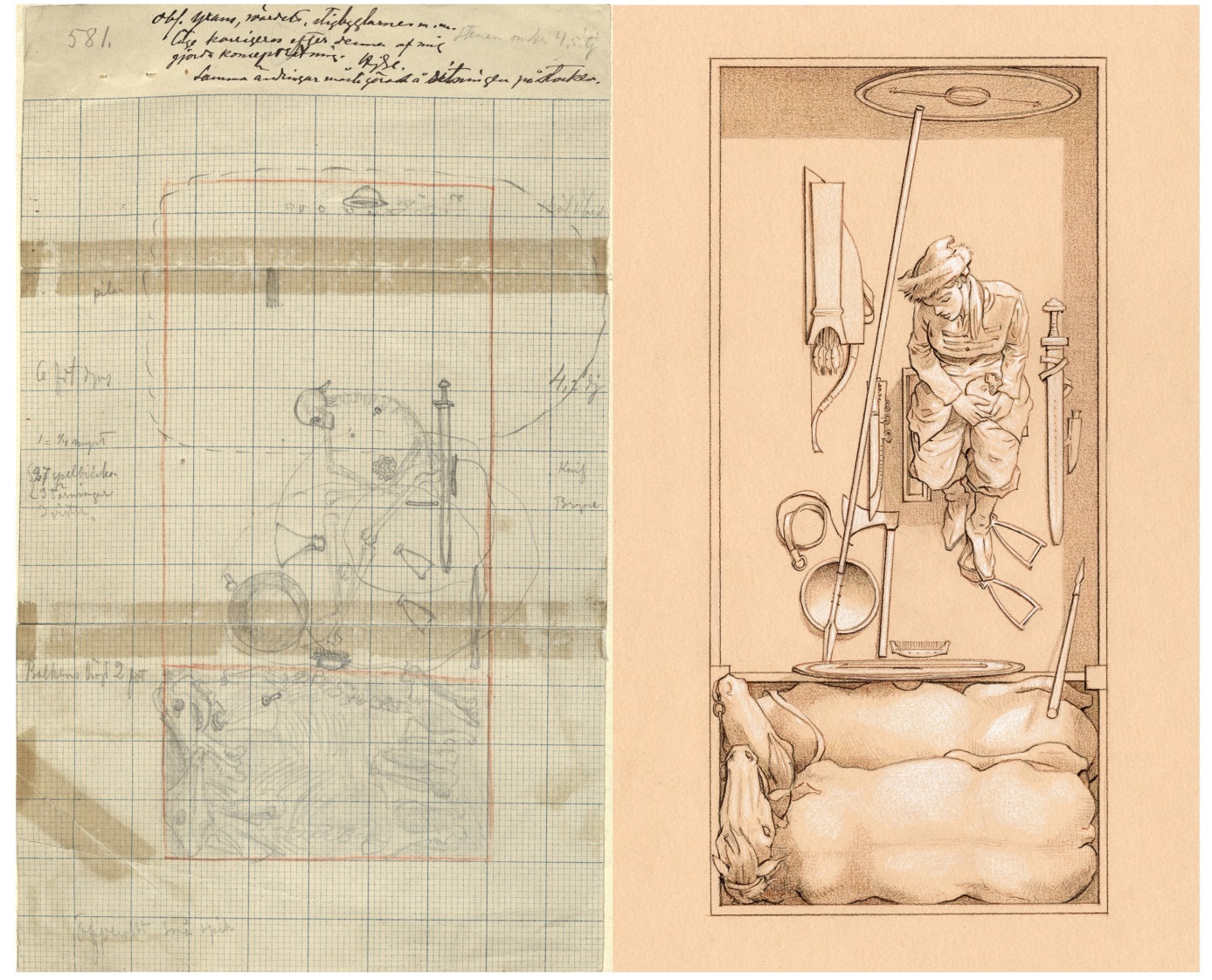
**

**Figure S1. 1a and 1b**. The original field documentation of grave Bj 581, executed by excavator Hjalmar Stolpe in 1877 and kept in Antikvariska Topografiska Arkivet (ATA) in Stockholm, Sweden (available online https://share.mediaflowpro.com/?PZAMDNU8TG). The positioning of the skeleton in the grave induced an interpretation of the deceased as have being seated in the grave. Reconstruction by Þórhallur Þráinsson, © Neil Price.

**
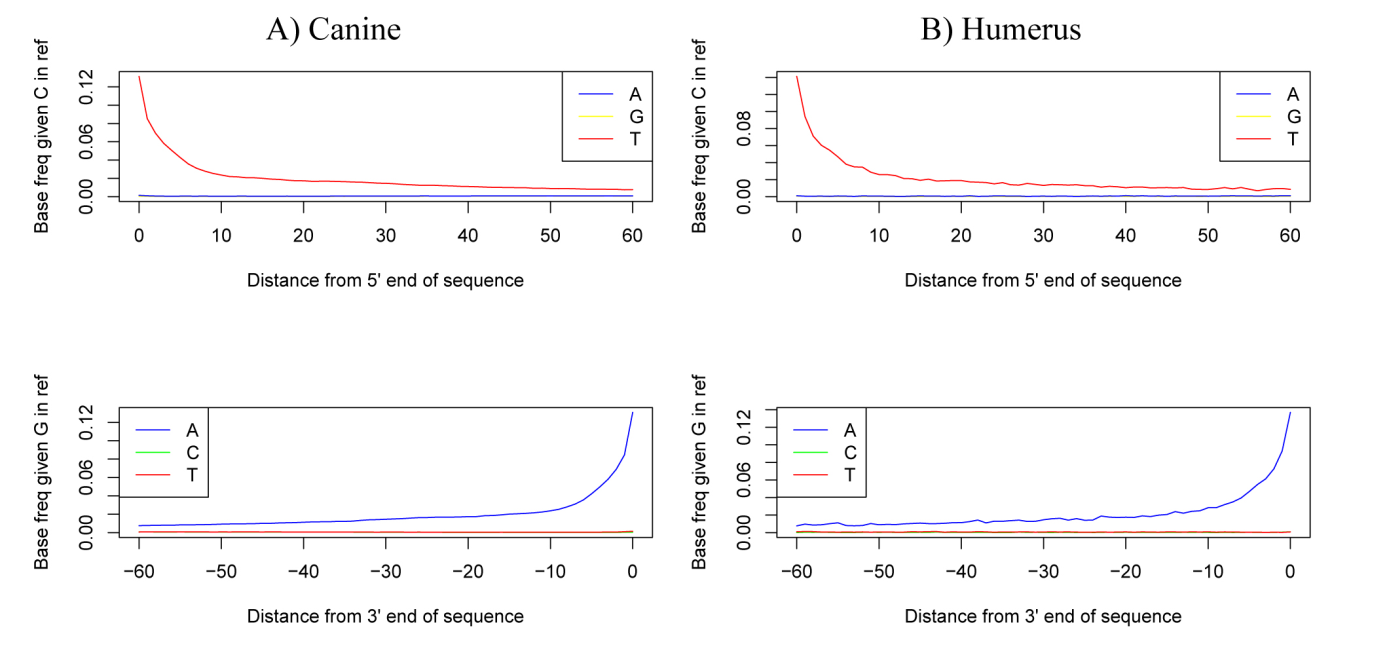
**

**Figure S4. 1a and 1b.** Nucleotide misincorporation patterns observed in the two libraries and their distance in bp from the 3’ and 5’ ends of DNA sequence fragments.

**
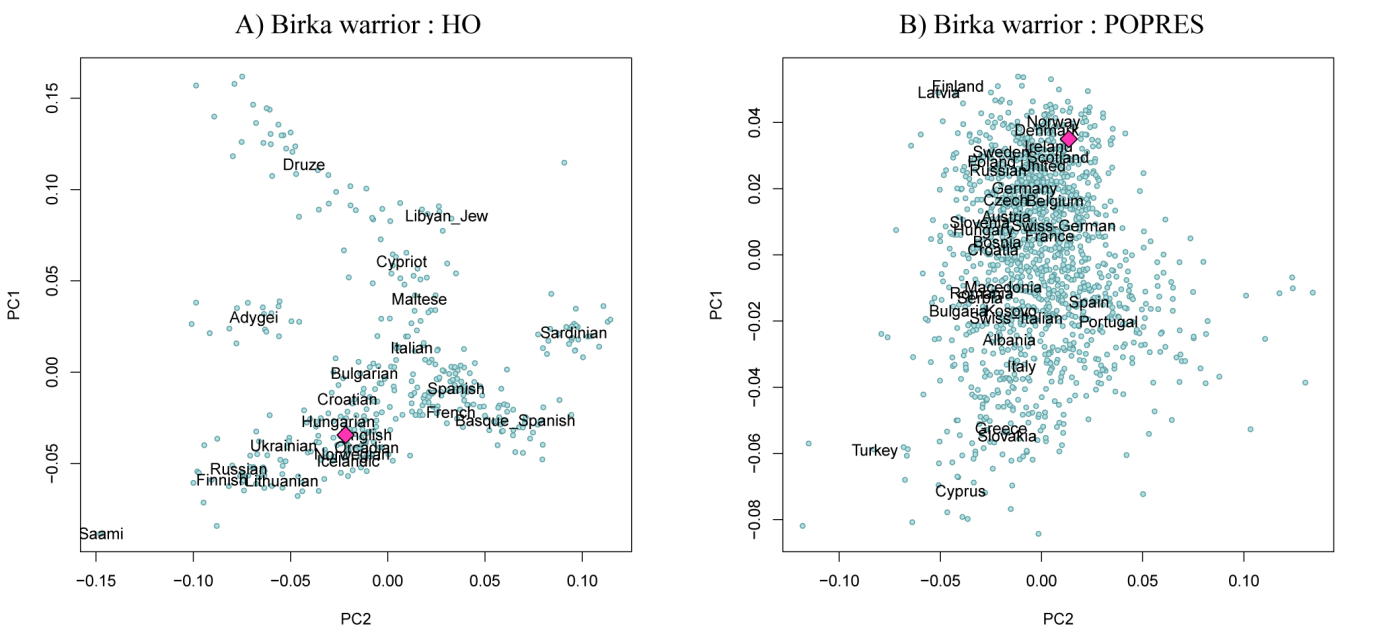
**

**Figure S4. 2a and 2b.** PCA plots the Birka warrior (brk581a, Bj581) with European reference panel populations from: A) Human Origins (HO) (Patterson et al., 2012; Lazaridis et al., 2014) and B) Population Reference Sample (POPRES) (Nelson et al., 2008).


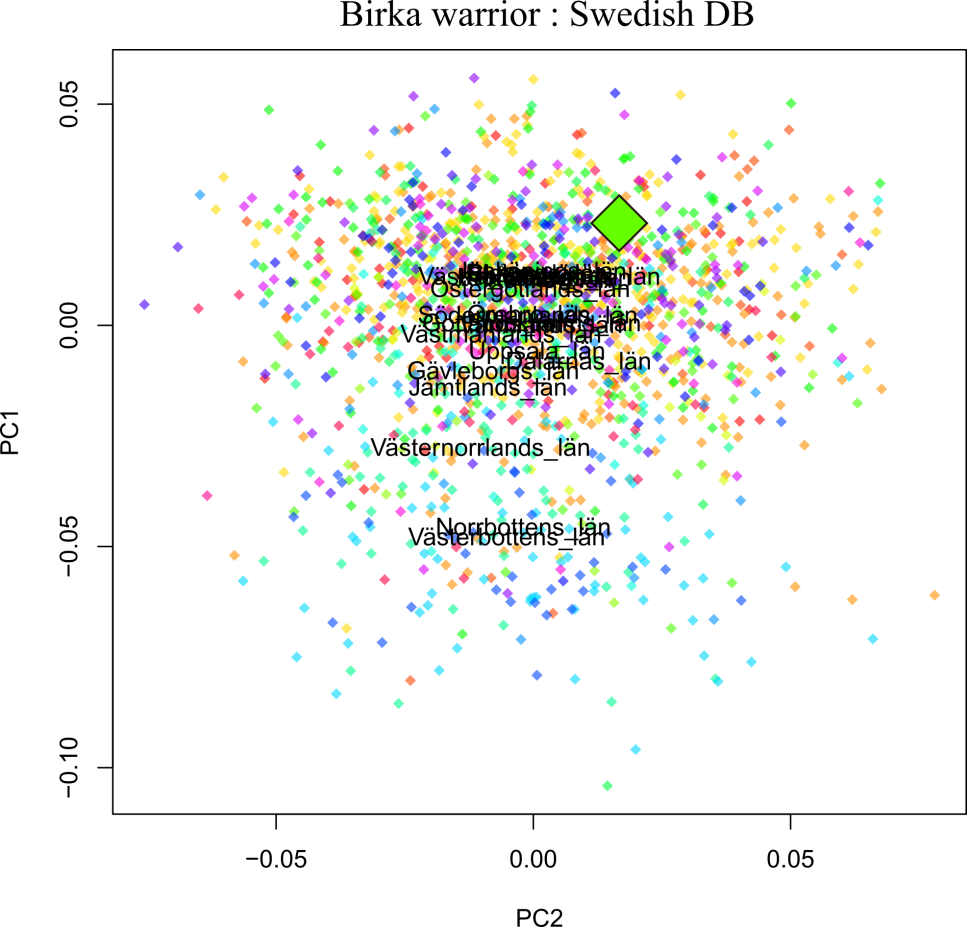


**Figure S4. 3.** A PCA plot the Birka warrior (brk581a, Bj581) with the Swedish reference panel (Salmela et al., 2011).

**
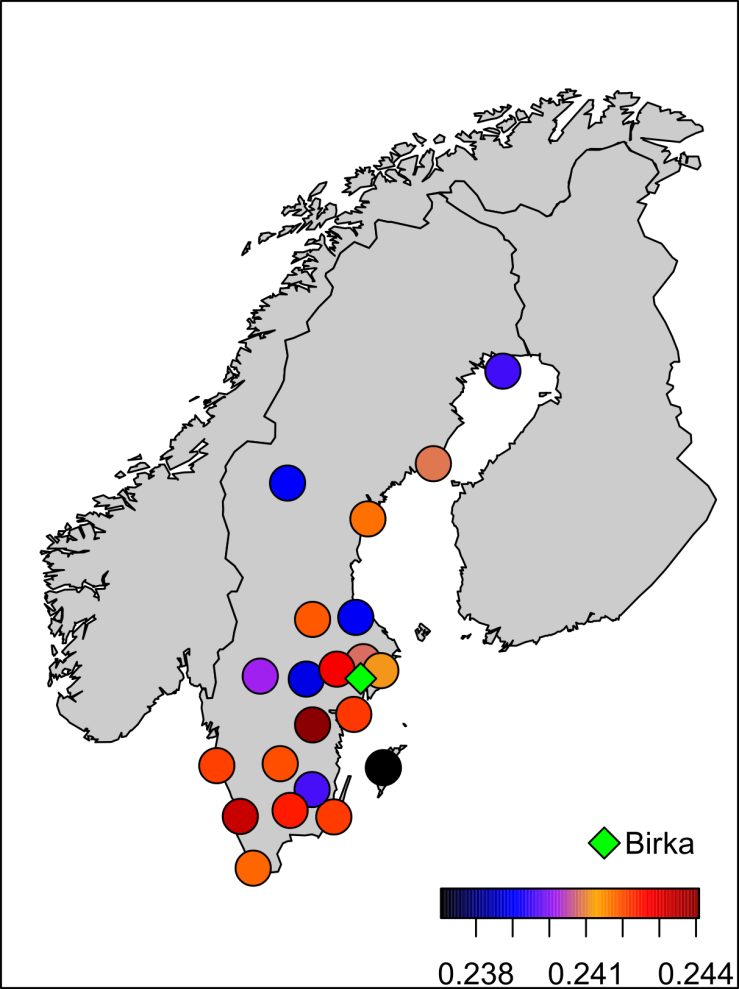
**

**Figure S4.4.** A map visualising the results of *f_3_*-statistic in which the individual from grave Bj 581 was compared to a Swedish population reference panel (Salmela et al., 2011) consisting of 1525 individuals grouped into 21 subpopulations representing Swedish counties.

**
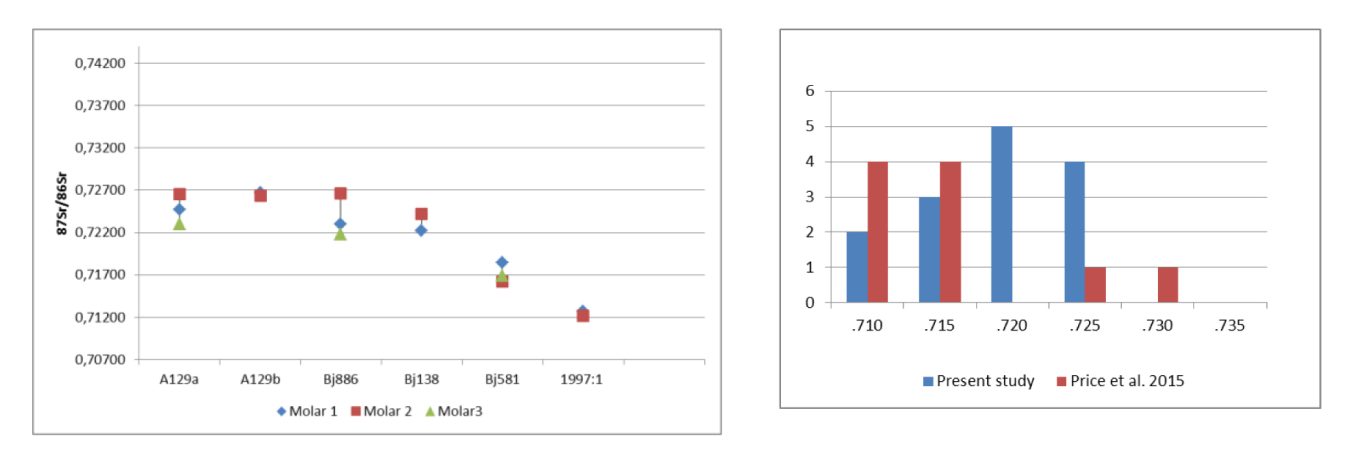
**

**Figure S5. 1a and 1b.** The distribution of strontium values between individuals and teeth and the variation in strontium isotopic range (87Sr/86Sr) between teeth of Birka individuals. Data in Table S4.1 and Price et al. (Price et al., 2015).


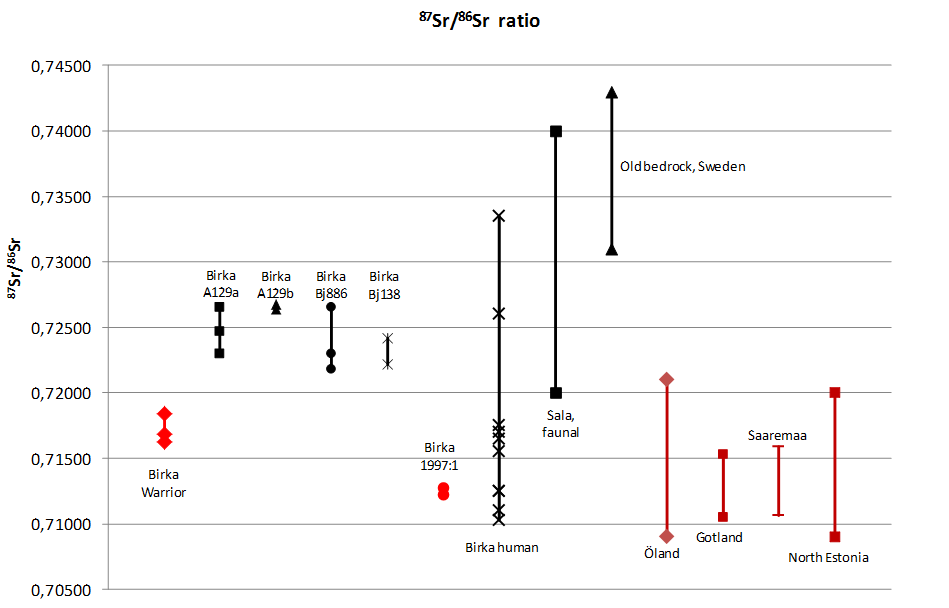


Figur S5.2. Strontium data from: Birka human, Price et al 2015; Sala faunal, Bäckström and Price 2016; Old bedrock, Sweden, Eriksson et al. 2016; Öland faunal, Wilhelmson and Ahlström 2015; Gotland faunal, Peschel et al 2017; Saaremaa and North Estoniafaunal, Oras et al. 2016.
